# Recommendations for guidelines for promoting mental health in the workplace: an umbrella review

**DOI:** 10.1590/0034-7167-2024-0086

**Published:** 2024-12-16

**Authors:** Evelin Daiane Gabriel Pinhatti, Amanda Salles Margatho do Nascimento, Regina Celia Bueno Rezende Machado, Rosangela Aparecida Pimenta, André Estevam Jaques, Maria do Carmo Fernandez Lourenço Haddad

**Affiliations:** I Universidade Estadual de Londrina. Londrina, Paraná, Brazil; II Universidade de São Paulo. Ribeirão Preto, São Paulo, Brazil; III Universidade Estadual de Maringá. Maringá, Paraná, Brazil

**Keywords:** Mental Health, Workplace, Systematic Review, Health Promotion, Occupational Health., Salud Mental, Lugar de Trabajo, Revisión Sistemática, Promoción de la Salud, Salud Laboral.

## Abstract

**Objectives:**

to summarize the recommendations of guidelines for promoting mental health in the workplace.

**Methods:**

an umbrella review, according to Joanna Briggs Institute and Preferred Reporting Items for Systematic reviews and Meta-Analyses methodological assumptions. Data collection was carried out in January 2021 and updated in July 2023 in the American Psychological Association, Cochrane Library, EMBASE, National Library of Medicine, and Scopus databases. Systematic reviews that assessed guidelines with recommendations for mental health care for workers were included. PROSPERO registration CRD42023461845.

**Results:**

four systematic reviews published between 2015 and 2018 were identified. The abstracts highlighted actions that facilitate and inhibit the recommendations as well as three categories of intervention: primary prevention - worker protection; secondary prevention - promoting workers’ mental health; and tertiary prevention - supporting, monitoring and rehabilitating workers upon returning to work.

**Conclusions:**

the interventions are based on prevention, promotion and early recognition, support and rehabilitation of mental health problems.

## RESUMEN

**Objetivos:**

resumir las recomendaciones de las guías para promover la salud mental en el lugar de trabajo.

**Métodos:**

revisión general, según los supuestos metodológicos del *Joanna Briggs Institute y Preferred Reporting Items for Systematic reviews and Meta-Analyses*. La recolección de datos se realizó en enero de 2021 y se actualizó en julio de 2023, en las bases de datos *American Psychological Association, Cochrane Library*, EMBASE, *National Library of Medicine y Scopus*. Se incluyeron revisiones sistemáticas que evaluaron guías con recomendaciones para el cuidado de la salud mental de los trabajadores. Matrícula PRÓSPERO CRD42023461845.

**Resultados:**

se identificaron cuatro revisiones sistemáticas publicadas entre 2015 y 2018. Los resúmenes destacaron acciones facilitadoras e inhibidoras de las recomendaciones, así como las tres categorías de intervención: prevención primaria - protección del trabajador; prevención secundaria - promover la salud mental de los trabajadores; y prevención terciaria: apoyo, seguimiento y rehabilitación del trabajador al regresar al trabajo.

**Conclusiones:**

las intervenciones se basan en la prevención, promoción y reconocimiento temprano, apoyo y rehabilitación de los problemas de salud mental.

## INTRODUCTION

Mental health is determined by a complex interaction between individual, social and structural stresses and vulnerabilities^(1)^. Within these interactions, work can be a protective factor or a generator of harm to mental health^(2)^. It is estimated that 15% of working-age adults experience some type of mental disorder at some point during their working lives^(3)^.

The World Health Organization (WHO) warns that mental illness is among the main causes of incapacity at work, recommending effective actions to improve workers’ mental health, fulfilling their right to a safe and healthy workplace^(3)^.

The proposal for the Global Agenda for Sustainable Development Goals (SDGs), determined by the United Nations summit, has goals and targets to be met by 2030. In its 8^th^ goal, it describes the valorization of decent work for economic growth, aiming at structural and functional characteristics of work that do not harm workers’ health^(4)^.

To care for mental health, managers must create and maintain a mentally healthy workplace. Combined workplace mental health actions can be used in this approach, with guiding principles anchored in: protecting mental health by reducing work-related risk factors; promoting mental health by developing the positive aspects of work and workers; and addressing mental health problems among workers^(5)^.

These actions must be carried out both in the occupational environment and directly with the workers involved, and have been classified as integrated strategies between primary, secondary and tertiary prevention methods^(5-7)^.

Primary stress prevention is a proactive approach, mainly focused on the workplace and workers, before they show any symptoms related to mental illness. Secondary stress prevention is aimed at modifying workers’ reaction to the problem that presents itself at an early stage, but which has an impact on psychological health. Tertiary care is reactive, as mental health problems occur, involving treatment of affected workers and support, rehabilitation and return-to-work^(6,7)^.

Workplaces are heterogeneous, complex and dynamic, and carrying out integrated preventive actions is a challenge for everyone involved^(6)^. Therefore, institutional strategies focusing on one level of prevention can be adopted; however, coordination between these levels is relevant, as failures in primary prevention require secondary and/or tertiary prevention efforts^(7)^ that could be avoided.

In this context, nursing plays a crucial role in strategies to promote workers’ health, identifying occupational risks, actions and guidance on lifestyle, and other workers’ health needs. Understanding the specificities and limitations of the production process also enables nurses to expand their actions with workers, aiming to transform working conditions and promote health effectively^(8)^.

To establish prevention measures in the workplace, recommendations indicated by guidelines can be used, which are statements that provide recommendations intended to guide decision-making in a given area based on scientific evidence published, mainly in systematic reviews (SRs)^(9)^.

SRs are a rigorous scientific approach to assess and summarize evidence related to specific questions, which can assist in decision-making^(10)^. There has been an exponential increase in the publication of SRs. It is worth noting that approximately 22 new SRs are published every day^(11)^. Thus, SR summaries emerged as a methodology to systematically group, assess and synthesize the results of several SRs called umbrella reviews^(10)^.

There is growing recognition of the importance of mental health issues in the workplace, but there is a need to adopt effective, evidence-based mental health actions and interventions^(12)^. Although mental illness is influential on workers’ health, a preliminary search carried out in the Joanna Briggs Institute Evidence Synthesis and the Cochrane Library found no comprehensive reviews that summarized evidence on recommendations for promoting workers’ mental health.

Given the above and the complexity of the approach to mental health in the workplace and workers’ and managers’ incipient knowledge about the recommendations that guide institutions for formulating institutional policies, this study had as its research question: what are the recommendations of guidelines for promoting mental health in the workplace?

## OBJECTIVES

To summarize the recommendations of guidelines for promoting mental health in the workplace.

## METHODS

### Study design

This study consists of an umbrella review, which has as its main focus to summarize the results of existing research related to a given topic or issue^(10)^. It can also be referred to as an overview, general SR review or comprehensive review. This review was developed according to the Joanna Briggs Institute (JBI) methodology for umbrella review and Preferred Reporting Items for Systematic reviews and Meta-Analyses (PRISMA) recommendations^(10,13)^. The study was registered in the International Prospective Register of Systematic Reviews (PROSPERO) database - Protocol CRD42023461845.

### Review question

To search for studies and formulate the research question, the PICo strategy was used, a mnemonic for “Population” (workers), “Phenomenon of Interest” (recommendations of guidelines for mental health promotion) and “Context” (workplace). Thus, the following research question was established: what are the recommendations of guidelines for promoting workers’ mental health in the workplace?

### Inclusion criteria

Full-text available SR studies that identified and assessed guidelines for promoting mental health in the workplace and that addressed organizational, individual or integrated interventions were included.

### Research strategy

The search for SRs took place in January 2021, and the update, in July 2023, in five databases, such as Cochrane Library - Wiley, PubMed - National Library of Medicine, Scopus - Elsevier, EMBASE - Elsevier and American Psychological Association (PsycINFO) - EBSCO.

For the search strategy, the controlled descriptors “mental health”, “workplace”, “guideline” and “systematic review” were used, associated with the Boolean operator “AND”, adapting to the descriptors specific to each database. According to the JBI Manual for Evidence Synthesis recommendation, “systematic review” was included in the descriptors, considering that some databases do not have a predefined search filter for review articles^(10)^. There was no language or time delimitation.

### Study selection

**Figure 1:**
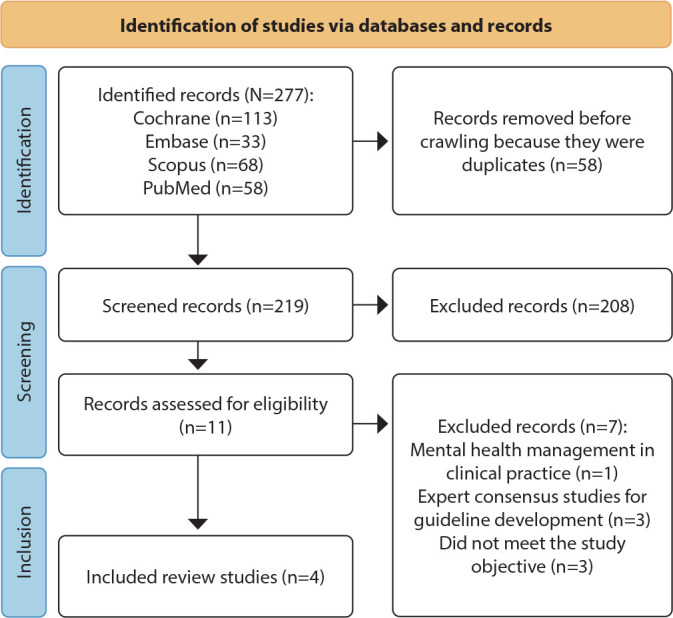
Stages for identifying and selecting systematic reviews on guideline recommendations for promoting mental health in the workplace, Londrina, Paraná, Brazil, 2023

The process of identifying and selecting SR studies occurred in four stages, following the recommendation according to the PRISMA items. In the first, the records were identified in the databases; in the second, the records were selected by reading titles and abstracts; in the third, record eligibility was assessed according to the research question; and in the fourth, SR studies were included, as shown in Figure 1. Moreover, the reference list of identified and included SRs was also examined to identify any other SRs not found in the initial search of the electronic databases^(13)^.

To reduce research bias, records were examined by two independent reviewers, after inspecting titles and abstracts as well as reading the full text of articles. Disagreements were resolved by consensus and, in case of doubts, a third reviewer was consulted. To organize and manage the data, an instrument developed in Microsoft Excel^®^ version 2021 was used, which included information regarding the title of studies, year of publication, whether it answered the research question and the reason for exclusion of the study. A detailed screening of studies was also performed in this software, and duplicates were removed.

### Quality assessment of systematic reviews

In order to examine the quality of included SRs, the JBI critical appraisal checklist for SRs and research syntheses was used. The checklist includes 11 items with response options of “yes”, “no”, “not clear” and “not applicable”^(10)^. Data were extracted by one researcher and checked by a second researcher. Discrepancies were resolved by consensus.

### Data extraction from systematic reviews

A customized version of the JBI Manual for Evidence Synthesis data extraction tool was used^(10)^. Thus, the following characteristics were collected: 1) author/year; 2) objectives of included SR; 3) setting and context; 4) number of databases searched; 5) number of guidelines included in each SR; 6) publication date range of guidelines included in SR; 7) instrument used to assess the quality of the guidelines; and 8) main results reported.

### Summary of included systematic reviews

The results of interest extracted from the included SRs are presented in a narrative format. Figures, charts and tables were included, when appropriate, to assist in data presentation. To present the summary of the evidence, a visual indication was used, in which facilitating actions are highlighted in green and inhibitory actions in red, to promote mental health in the workplace, as recommended by the JBI Manual for Evidence Synthesis^(10)^.

Recommendations for promoting mental health in the workplace involve three levels of prevention. In this study, primary prevention recommendations are those interventions that aim to prevent work-related mental health problems, with the aim of reducing work-related stressors, or related to workplace structures^(14)^.

Secondary prevention recommendations were those that target workers, with the aim of improving their ability to cope with and resist workplace stressors as well as preventing the progression of subclinical mental health problems to diagnosable disorders^(14)^. Tertiary prevention was defined as that which involves the treatment of workers with mental health issues and support and rehabilitation for work^(14)^.

## RESULTS

Initially, this research identified 277 articles and, after selecting the studies, four SRs were selected^(15-18)^, constituting this umbrella review. Furthermore, the list of references of included SRs was read, however no study was selected, as shown in Figure 1.

Table 1 presents the overall quality of included SRs, with all obtaining a score of 100% in items 2-5 and 11, 75% in items 6-8 and 10, and item 1 obtaining 50% adequacy. Item 9, which assesses the risk of bias, was not met in any of the studies.

**Table 1:** Quality assessment of included systematic reviews using the Joanna Briggs Institute tool, Londrina, Paraná, Brazil, 2023

JBI items	Author/year				Percentage (%) of criteria met
	Joosen *et al*., 2015^(15)^	Dewa *et al.*, 2016^(16)^	Memish *et al.*, 2017^(17)^	Nexo *et al.*, 2018^(18)^	
1	S	NC	S	NC	50
2	S	S	S	S	100
3	S	S	S	S	100
4	S	S	S	S	100
5	S	S	S	S	100
6	NC	S	S	S	75
7	NC	S	S	S	75
8	S	S	NC	S	75
9	N	N	N	N	0
10	S	S	NC	S	75
11	S	S	S	S	100

*Y - Yes; N - No; NC - Not clear; NA - Not applicable;*

*JBI tool items: 1. Is the review question clearly and explicitly stated? 2. Were the inclusion criteria appropriate for the review question? 3. Was the search strategy appropriate? 4. Were the sources and resources used to search for studies appropriate? 5. Were the study assessment criteria appropriate? 6. Was critical appraisal conducted by two or more reviewers independently? 7. Were there methods to minimize errors in data extraction? 8. Were the methods used to combine studies appropriate? 9. Was the likelihood of publication bias assessed? 10. Were the recommendations for policy and/or practice supported by the reported data? 11. Were specific guidelines for new research appropriate?*

The authors of selected SRs performed searches in between four and 11 search sites. The guidelines were found in repositories of occupational health and safety organizations, government agencies^(16,17)^, guideline databases^(15,16,18)^ and Google^®(15-18)^ and databases such as PubMed, PsycNET, Web of Science and Arts & Humanities Citation Index^(15,18)^.

The guidelines were developed for workplaces, employers and employees, and were prepared from 2003 to 2015. The number of guidelines included in SRs ranged from three to 20 guidelines, totaling 54 guidelines. To assess guideline quality, the reviews used the Guidelines for Research and Evaluation II (AGREE II).

Chart 1 presents the characteristics of the SRs that assessed guidelines for promoting mental health in the workplace in relation to author, year of publication, objectives, number of guidelines included, AGREE II assessment and development country or organization of the guidelines.

**Chart 1:** Characteristics of systematic reviews that assessed guidelines for promoting mental health in the workplace in relation to author, year of publication, objectives, number of guidelines included and development country or organization of the guidelines, Londrina, Paraná, Brazil, 2023

Author/year	Objectives	Included guidelines	AGREE II^*^	Development country or organization of the guidelines
Joosen *et al.*, 2015^(15)^	Identify occupational health guidelines focused on the management of mental disorders and stress-related symptoms from different countries around the world, and describe them, compare their content and assess their quality of development and reporting.	14 guidelines	Mean AGREE II 61.5%	Netherlands, United Kingdom, Japan, Finland and Republic of Korea
Dewa *et al.*, 2016^(16)^	Identify best practice guidelines for mental illness-related disability for employers in grey literature.	Three guidelines	Mean AGREE II 72.2%	Canada, United Kingdom and Australia
Memish *et al.*, 2017^(17)^	Determine the quality of workplace mental health guidelines; assess the comprehensiveness of recommendations addressing the three segments of integrated approach: preventing harm and minimizing risk factors in the workplace; promoting positive and protective factors in the workplace; and managing mental health problems, regardless of cause.	20 guidelines	Mean AGREE II 59.8%	Canada, United Kingdom, Australia, European Union, World Health Organization
Nexo *et al.*, 2018^(18)^	Systematically review the quality of development of guidelines that provide recommendations for workplace stakeholders to prevent work-related mental illness, detect and manage early signs, or facilitate return-to-work; and compare the content of recommended interventions included in the guidelines and the reported evidence supporting the expected outcomes of interventions.	17 guidelines	Mean AGREE II 54.5%	Australia, Canada, Denmark, England, New Zealand, Sweden, Organization for Economic Cooperation and Development, World Health Organization

* *Percentage of adequacy according to Guidelines for Research and Evaluation II.*

Chart 2 presents a summary of evidence based on the characteristics presented in SR. In the summarized findings, facilitating actions are highlighted in green and inhibitory actions in red for promoting mental health in the workplace.

**Chart 2:** Synthesis of recommendations presented in systematic reviews for promoting mental health in the workplace, Londrina, Paraná, Brazil, 2023

Phenomena of interest	Authors	Synthetised discovery
Management of mental disorders and stress-related symptoms.	Joosen *et al.*, 2015^(15)^	Assessment of workplace factors relevant to mental health. Assessment of work competencies and skills. Assessment of workload, stressors and job content. Assessment of communication and/or problem-solving skills between worker and supervisor. Assessment of workers’ mental health symptoms. Examining factors that influence recovery in private and professional life. Recommendations on coping strategies (communication and problem-solving skills). Assessment of risk of self-harm/suicide. Early onset counselling, guidance and support. Specific mental health treatment psychological interventions, cognitive and behavioral interventions, self-management strategies, return-to-work interventions, work adaptations with reduction of stressful conditions and/or reduction of working hours and demands, prohibition of night shifts, advice to the employer to maintain contact with workers and give instructions to coworkers to avoid stigma. Monitoring of workers by leaders and other professionals involved in the recovery process and assessment of work capacity.
		Drug treatment indicated only for severe mental disorders or insomnia.
Mental illness-related disability best practices for employers.	Dewa *et al.*, 2016^(16)^	Development of organizational policies/procedures. Establishment of a supportive work environment. Establishment of a disability leave plan with regular communication between the organization and the employee. Return-to-work coordinated, planned, and facilitated by a designated coordinator who supports communication between the leader, the employee, and the organization. Provision of intensive, multidisciplinary, evidence-based interventions. Support for workers to access available treatments. Recommendation of training for leaders. Provision of mental health awareness and training for all workers to avoid stigma. Assessment of the work performed by workers and supervisors.
		Clinical interventions without changes in work and/or professional guidance.
Recommendations addressing the three segments of the integrated approach to managing mental health problems.	Memish *et al.*, 2017^(17)^	Recommendations for organization to minimize risk factors and promote positive factors at work. Recommendations for preventing mental illness targeted at the individual level. Development of positive leadership styles, climate or organizational culture in the workplace. Identification and treatment of mental health problems in the workplace.
		Lack of resources and/or trained personnel to implement interventions.
Recommendations for preventing, detecting and managing early signs of work-related mental illness or facilitating return-to-work.	Nexo *et al.*, 2018^(18)^	Mental health policy at all levels of the organization (worker involvement, planning, resources, roles and responsibilities). Identification and elimination of psychosocial risks on an ongoing and systematic basis. Improvement of job design and person-job fit through skills training and rotating work schedules in locations where psychosocial risks cannot be eliminated. Implementation of educational programs to raise awareness of mental health and avoid stigma associated with mental illness. Encouragement of positive work factors, such as peer support and rewards. Increase in communication and relational skills among leaders to prevent mental illness. Assessment of workers’ mental health. Strategies for managing stress and mental health problems. Provision of workplace counseling and adjustments for workers with mental illness. Encouragement of support/guidance among coworkers. A coordinated and multidisciplinary return-to-work approach. Training of leaders to detect signs of mental illness.
		Inadequate work environment management, such as moral harassment and conflicts at work. Poorly organized work with high demands, little control and threats of violence from clients.

The SRs presented the recommendations of guidelines for mental health in the workplace at primary level^(15,17,18)^, secondary level^(15,17,18)^ and tertiary level^(15-18)^. In guideline assessment, the predominance of integrated recommendations between the levels of prevention (74%) stood out.

**Figure 2:**
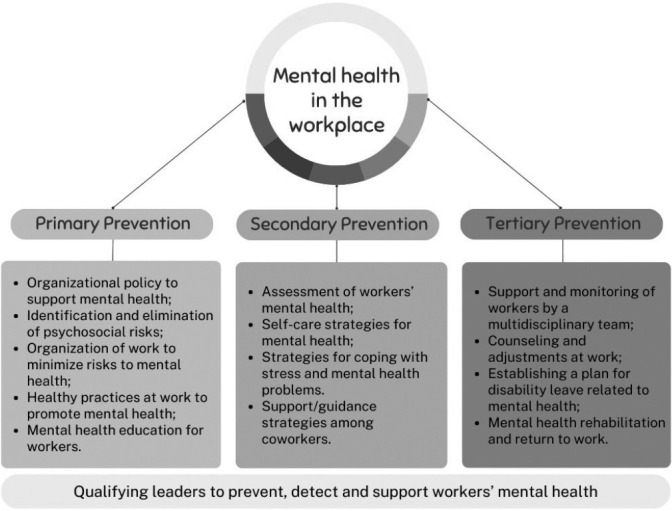
Summary of prevention levels of the main recommendations identified in systematic reviews that assessed guidelines for promoting mental health in the workplace, Londrina, Paraná, Brazil, 2023

Figure 2 summarizes the main recommendations presented in the grouped SR, according to the level of prevention, as follows: 1) Primary prevention recommendations - worker protection; 2) Secondary prevention recommendations - detecting and promoting workers’ mental health; and 3) Tertiary prevention recommendations - supporting, monitoring and rehabilitating workers upon returning to work after absence. Training of leaders was recommended for the three levels of prevention.

## DISCUSSION

It was observed that the recommendations of the guidelines identified in this study for promoting mental health in the workplace are based on mental health protection and promotion, early recognition of problems, support and rehabilitation of workers upon returning to work.

When approaching mental health, it is important to understand that each organization has its own needs, and this will require a set of strategies to be defined and addressed by a comprehensive health program. Therefore, it is necessary to encourage the cooperation and participation of employers, leaders and workers, in order to hold them accountable and mobilize the knowledge necessary to obtain better results in mental health^(19)^.

Regarding guideline assessment, the AGREE II, used in the reviews, offers the opportunity to systematically, specifically and objectively assess the quality of the guidelines listed in the studies analyzed. However, although this instrument is used to assess guideline rigor and transparency, it does not distinguish between high and low quality. The subjectivity of the assessments is also highlighted, with evaluators being able to interpret the items and the scores in different ways^(9)^.

In line with the results found, during the design of this study, the WHO published guidelines for mental health at work, which supported the findings of this research. The WHO guidelines emphasized that the approach to mental health should be broad and comprehensive, and that it is important for workplaces to adopt a scenario conducive to change, with a commitment to combat stigma and discrimination, coordinating multisectoral and participatory approaches for effective interventions^(20)^.

The shared participation of employers, managers and workers is essential for developing a healthy work environment. In this context, it is imperative to provide information on how to reduce risks that potentially cause harm and how to enhance workplace factors that promote psychological health, constituting the primary prevention of mental health problems^(21)^.

Assessing workers’ mental health and well-being is a key factor in assessing the organization’s needs. This may include assessing mental health and well-being indicators, level of mental health knowledge, and identifying sources of stress in the workplace. Based on a thorough assessment of the workplace, specific initiatives can be implemented to minimize risks and/or meet the organization’s needs, in order to prevent or reduce harm to workers’ mental health^(22)^.

A workplace that prioritizes actions to protect mental health must mitigate work-related aggravation factors, with identification, control and elimination of risks that can be eliminated and assessment and control of risks that cannot be eliminated. It is necessary to establish policies that reduce tension in the workplace, building a positive organizational climate^(21)^.

Institutional policy must be based on respect, consideration, tolerance, recognizing diversity, addressing disrespectful behavior, violence, discrimination, harassment and incivility with severity, thus contributing to the organizational culture representing the institution’s shared values^(22)^.

Work configuration is recognized as one of the risk factors that cause psychological impact on workers. Therefore, it is necessary to analyze the way in which tasks are organized, ensure that the workload is aligned with workers’ capabilities and resources, balance demands with the control that workers have over their work, offer opportunities for career development, improve communication and, if possible, make the work schedule more flexible^(22)^. Other actions include ensuring work-life harmony, providing more autonomy over how work is done, respecting boundaries between work and rest hours, involving them in decision-making, building a culture of recognition, and providing a decent wage^(23)^.

Assignment of workers’ activities should also be considered, and it is advisable to match a worker’s psychological skills with the job demands. Another strategy that the workplace can offer individuals in order to attract, retain and develop workers is to provide opportunities for growth and development, to enhance their skills, and to ensure that they obtain relevant feedback on their performance^(21,23)^. Interventions involving participatory approaches can be considered to obtain better results in reducing emotional distress^(20)^.

Regarding recommendations for secondary prevention, promoting workers’ health can be integrated into prevention initiatives. In addition to the actions carried out by the organization, it is necessary for workers to also take responsibility for taking care of their mental health, associating the promotion of preventive approaches and multiplying the benefits^(7)^.

For workers to understand the importance of preventive measures, mental health knowledge and awareness are necessary. This requires basic information, explaining what a mental health problem is, the signs and symptoms and common mental health problems, what to do if one needs support or has a colleague who needs help^(7)^. Understanding mental health makes it easier to recognize, identify and manage psychological problems, helping to reduce stigma and raise awareness of self-care options^(7,20)^.

Strategies for establishing self-care behaviors include healthy eating, sleeping well, participating in relaxing activities, engaging with family and friends, developing healthy habits, not smoking or abusing alcohol, cultivating healthy relationships, and practicing physical activity^(7,24)^.

In order to identify mental health problems, investigation is necessary^(7,20)^, which can be done through self-reported questionnaires. The relevance of this strategy is based on the identification and approach of psychological health problems when they are in their initial stage^(25)^. The recommendations do not provide evidence of potential benefits or harms from worker screening interventions^(20)^. Therefore, monitoring the mental health status, in addition to allowing workers to self-assess, can contribute to institutional planning.

Individual interventions can be used to prevent and manage psychological distress, limiting the onset of more serious mental health problems. Interventions can be delivered in person or digitally, through apps or online^(20)^.

Strategies focus on interpersonal skills, stress management, conflict resolution, problem solving, communication, time management, mindfulness, resilience, positive coping skills, relaxation techniques, physical activity, and lifestyle interventions^(7,20,24)^. Psychological interventions based on cognitive behavioral therapy, acceptance and commitment therapy and positive psychology are also used^(20)^.

Promoting mental health at work is a complex task and involves building capacity, raising awareness and providing opportunities to recognize and act early on mental health conditions^(20)^.

In tertiary prevention recommendations, in cases of established mental illness, actions can be taken to reduce the distress and dysfunction associated with a mental disorder. Actions may involve ensuring immediate access to appropriate treatment, providing improved quality of life and engagement at work, contributing to recovery from mental health problems^(26)^.

Early intervention is essential in managing mental health problems. To this end, available support services should be made known to workers, and the benefits of professional care should be explained^(27)^. Individual interventions can also be considered to reduce symptoms and improve work effectiveness.

In cases where there is a need to take time off work, interventions are required for rehabilitation and return-to-work^(20)^. A meta-analysis study that assessed these interventions identified that the strategies are based on coping mechanisms, stress management, relaxation techniques, and sharing of personal experiences with work-related stressors. Coordination interventions are also carried out with case management, education and training, workload assessment, improved communication, and increased accessibility between workers and their supervisors. It is also reported that case management composed of multidisciplinary teams and the adoption of combined strategies are more effective^(28)^.

Return-to-work initiatives should aim to adapt work environments to match workers’ capabilities, needs and preferences, and should be tailored to each worker. Support for rehabilitation and return-to-work provides greater chances of achieving positive outcomes, with reduced chances of recurrence of mental disorders^(20)^.

In addition to the forms mentioned, there are integrated recommendations that cover coordination between the three levels of prevention, making it possible to carry out prevention actions in synergy^(7)^. Organizations should normalize and support mental health, making mental health and well-being a priority, and offering support and prevention services. Leaders and managers across the organization should be supported to create a culture of mental health care^(23)^.

Leadership plays a fundamental role at all levels of prevention, whether in developing a mentally healthy work environment or in recognizing employees with psychological symptoms, acting across the board to improve mental health^(20,29)^.

To this end, managers and supervisors, i.e., leaders, need training to carry out early detection and response and support for return-to-work. Training may involve developing basic skills in people management, workflow and delegation skills that will contribute to mental illness prevention, such as how to approach and talk to a worker with a mental health condition and how to refer them to the help resources available in the organization^(29)^.

Furthermore, leaders should strive to reduce stigma associated with mental health issues and promote a workplace culture of transparency, trust, respect, openness, equality, inclusion, empathy and support rather than comparing and fostering a hostile environment of competition^(24)^.

For early detection and response, it is important that leaders know workers’ personalities and behaviors, in order to identify early on if there is any change in usual behavior^(29)^. Furthermore, developing leaders’ competency in mental health is essential and can improve workers’ knowledge, attitudes and help-seeking behavior^(20)^.

Leaders who do not prioritize institutional strategy and make demands on workers are also identified in the interventions, hindering the implementation and transition of learning to the workplace. Similarly, damaged relationships with workers are also highlighted as a barrier to the scope and sustainability of interventions^(27)^.

Scientific evidence recommends training leaders to support mental health^(20)^, since the influence of leadership is so relevant, as it can block or prevent the implementation of changes or undermine the effectiveness of any changes made^(29)^. Thus, awareness, stigma and stereotypes related to mental illness should be addressed with leaders, in order to reduce barriers to implementing interventions^(27)^.

Some factors can also interfere with the intervention process, including the lack of adequate training for those involved, insufficient time during the working day for participants, conflicting priorities, workload problems and lack of human resources coverage^(27)^.

A SR study that sought to identify the critical and success factors for implementing health and well-being practices at work identified as success factors regular communication between learning and governance structures that can facilitate local adaptations during the implementation of the strategy in the organization. Critical factors include the lack of continuity of efforts and adaptation of interventions, the level of involvement, support and organizational commitment to the intervention^(29)^.

Implementing strategies for a psychologically healthy workplace is complex. Therefore, a fundamental step is the assessment process, which can monitor, review and identify opportunities for improvement specific to each institutional reality^(5)^.

The SRs highlighted that guidelines with recommendations for mental health in the workplace are difficult to detect, being identified mainly through gray literature. Another relevant point is that the content and quality of guidelines varied significantly according to the AGREE II score, which may have occurred due to the assessment’s subjectivity as well as failure to meet some criteria at the time of assessment^(17,18)^.

### Study limitations

As limitations of this study, it is worth highlighting that the search was limited to scientific databases, not including sources from gray literature. Moreover, it is worth highlighting the reduced number of SRs found as well as the lack of SRs published in recent years. Furthermore, the SRs and guidelines assessed were prepared in developed countries. This finding demonstrates the need to expand the investigation and development of quality recommendations for promoting mental health in the workplace in underdeveloped countries. However, it is believed that this finding does not compromise the quality of the research, but indicates the relevance of future studies for developing recommendations and assessing worker health with the implementation of the recommendations of guidelines for promoting mental health.

### Contributions to nursing and health

The summary of the recommendations described in the guidelines assessed in relation to the quality of the formulation by SR contributes to dissemination of knowledge and can help managers and occupational nurses implement personalized strategies according to their reality, fulfilling the role of enhancing mental health promotion.

## CONCLUSIONS

The results of this umbrella review presented a synthesis of the recommendations for guidelines for promoting mental health in the workplace. The interventions are based on prevention, promotion and early recognition, support and rehabilitation of mental health problems. It is important to note that the guidelines constitute valuable tools for synthesizing and translating scientific evidence into recommendations, which can and should be used by managers in the workplace.

Based on the assumption that mental illness among workers is a growing public health problem, the workplace must be an environment that guarantees actions to prevent, promote, detect and manage mental health problems.
