# ERRATUM

**DOI:** 10.1590/0034-7167.20257801e03

**Published:** 2025-03-14

In the article “Recommendations for guidelines for promoting mental health in the workplace: an umbrella review”, with DOI number: https://doi.org/10.1590/0034-7167-2024-0086, published in Revista Brasileira de Enfermagem, 2024;77(6):e20240086, on page 5:

Chart 2 is replaced:

**Chart 2:** Synthesis of recommendations presented in systematic reviews for promoting mental health in the workplace, Londrina, Paraná, Brazil, 2023

Phenomena of interest	Authors	Synthetised discovery
Management of mental disorders and stress-related symptoms.	Joosen *et al.*, 2015^(15)^	Assessment of workplace factors relevant to mental health. Assessment of work competencies and skills. Assessment of workload, stressors and job content. Assessment of communication and/or problem-solving skills between worker and supervisor. Assessment of workers’ mental health symptoms. Examining factors that influence recovery in private and professional life. Recommendations on coping strategies (communication and problem-solving skills). Assessment of risk of self-harm/suicide. Early onset counselling, guidance and support. Specific mental health treatment psychological interventions, cognitive and behavioral interventions, self-management strategies, return-to-work interventions, work adaptations with reduction of stressful conditions and/or reduction of working hours and demands, prohibition of night shifts, advice to the employer to maintain contact with workers and give instructions to coworkers to avoid stigma. Monitoring of workers by leaders and other professionals involved in the recovery process and assessment of work capacity.
		Drug treatment indicated only for severe mental disorders or insomnia.
Mental illness-related disability best practices for employers.	Dewa *et al.*, 2016^(16)^	Development of organizational policies/procedures. Establishment of a supportive work environment. Establishment of a disability leave plan with regular communication between the organization and the employee. Return-to-work coordinated, planned, and facilitated by a designated coordinator who supports communication between the leader, the employee, and the organization. Provision of intensive, multidisciplinary, evidence-based interventions. Support for workers to access available treatments. Recommendation of training for leaders. Provision of mental health awareness and training for all workers to avoid stigma. Assessment of the work performed by workers and supervisors.
		Clinical interventions without changes in work and/or professional guidance.
Recommendations addressing the three segments of the integrated approach to managing mental health problems.	Memish *et al.*, 2017^(17)^	Recommendations for organization to minimize risk factors and promote positive factors at work. Recommendations for preventing mental illness targeted at the individual level. Development of positive leadership styles, climate or organizational culture in the workplace. Identification and treatment of mental health problems in the workplace.
		Lack of resources and/or trained personnel to implement interventions.
Recommendations for preventing, detecting and managing early signs of work-related mental illness or facilitating return-to-work.	Nexo *et al.*, 2018^(18)^	Mental health policy at all levels of the organization (worker involvement, planning, resources, roles and responsibilities). Identification and elimination of psychosocial risks on an ongoing and systematic basis. Improvement of job design and person-job fit through skills training and rotating work schedules in locations where psychosocial risks cannot be eliminated. Implementation of educational programs to raise awareness of mental health and avoid stigma associated with mental illness. Encouragement of positive work factors, such as peer support and rewards. Increase in communication and relational skills among leaders to prevent mental illness. Assessment of workers’ mental health. Strategies for managing stress and mental health problems. Provision of workplace counseling and adjustments for workers with mental illness. Encouragement of support/guidance among coworkers. A coordinated and multidisciplinary return-to-work approach. Training of leaders to detect signs of mental illness.
		Inadequate work environment management, such as moral harassment and conflicts at work. Poorly organized work with high demands, little control and threats of violence from clients.

With the color version of Chart 2:

**Chart 2:** Synthesis of recommendations presented in systematic reviews for promoting mental health in the workplace, Londrina, Paraná, Brazil, 2023

Phenomena of interest	Authors	Synthetised discovery
Management of mental disorders and stress-related symptoms.	Joosen *et al.*, 2015^(15)^	Assessment of workplace factors relevant to mental health. Assessment of work competencies and skills. Assessment of workload, stressors and job content. Assessment of communication and/or problem-solving skills between worker and supervisor. Assessment of workers’ mental health symptoms. Examining factors that influence recovery in private and professional life. Recommendations on coping strategies (communication and problem-solving skills). Assessment of risk of self-harm/suicide. Early onset counselling, guidance and support. Specific mental health treatment psychological interventions, cognitive and behavioral interventions, self-management strategies, return-to-work interventions, work adaptations with reduction of stressful conditions and/or reduction of working hours and demands, prohibition of night shifts, advice to the employer to maintain contact with workers and give instructions to coworkers to avoid stigma. Monitoring of workers by leaders and other professionals involved in the recovery process and assessment of work capacity.
		Drug treatment indicated only for severe mental disorders or insomnia.
Mental illness-related disability best practices for employers.	Dewa *et al.*, 2016^(16)^	Development of organizational policies/procedures. Establishment of a supportive work environment. Establishment of a disability leave plan with regular communication between the organization and the employee. Return-to-work coordinated, planned, and facilitated by a designated coordinator who supports communication between the leader, the employee, and the organization. Provision of intensive, multidisciplinary, evidence-based interventions. Support for workers to access available treatments. Recommendation of training for leaders. Provision of mental health awareness and training for all workers to avoid stigma. Assessment of the work performed by workers and supervisors.
		Clinical interventions without changes in work and/or professional guidance.
Recommendations addressing the three segments of the integrated approach to managing mental health problems.	Memish *et al.*, 2017^(17)^	Recommendations for organization to minimize risk factors and promote positive factors at work. Recommendations for preventing mental illness targeted at the individual level. Development of positive leadership styles, climate or organizational culture in the workplace. Identification and treatment of mental health problems in the workplace.
		Lack of resources and/or trained personnel to implement interventions.
Recommendations for preventing, detecting and managing early signs of work-related mental illness or facilitating return-to-work.	Nexo *et al.*, 2018^(18)^	Mental health policy at all levels of the organization (worker involvement, planning, resources, roles and responsibilities). Identification and elimination of psychosocial risks on an ongoing and systematic basis. Improvement of job design and person-job fit through skills training and rotating work schedules in locations where psychosocial risks cannot be eliminated. Implementation of educational programs to raise awareness of mental health and avoid stigma associated with mental illness. Encouragement of positive work factors, such as peer support and rewards. Increase in communication and relational skills among leaders to prevent mental illness. Assessment of workers’ mental health. Strategies for managing stress and mental health problems. Provision of workplace counseling and adjustments for workers with mental illness. Encouragement of support/guidance among coworkers. A coordinated and multidisciplinary return-to-work approach. Training of leaders to detect signs of mental illness.
		Inadequate work environment management, such as moral harassment and conflicts at work. Poorly organized work with high demands, little control and threats of violence from clients.

