# Supplementary material for: Recommendations for guidelines for promoting mental health in the workplace: an umbrella review
Source: Rev Bras Enferm. 2024 Dec 16;77(6):e20240086. doi: 10.1590/0034-7167-2024-0086 (PMC11654520; doi:10.1590/0034-7167-2024-0086)
Supplement: Translation

# Translation: Recomendações de diretrizes para promoção da saúde mental no local de trabalho: *umbrella review*

**DOI:** 10.1590/0034-7167-2024-0086pt

**Authors:** Evelin Daiane Gabriel Pinhatti, Amanda Salles Margatho do Nascimento, Regina Celia Bueno Rezende Machado, Rosangela Aparecida Pimenta, André Estevam Jaques, Maria do Carmo Fernandez Lourenço Haddad

**Affiliations:** I Universidade Estadual de Londrina. Londrina, Paraná, Brasil; II Universidade de São Paulo. Ribeirão Preto, São Paulo, Brasil; III Universidade Estadual de Maringá. Maringá, Paraná, Brasil

**Keywords:** Saúde Mental, Local de Trabalho, Revisão Sistemática, Promoção da Saúde, Saúde Ocupacional.

## Abstract

**Objetivos:**

sumarizar as recomendações de diretrizes para a promoção da saúde mental no local de trabalho.

**Métodos:**

*umbrella review*, conforme o pressuposto metodológico do *Joanna Briggs Institute* e *Preferred Reporting Items for Systematic reviews and Meta-Analyses*. A coleta de dados foi realizada em janeiro de 2021 e atualizada em julho de 2023, nas bases de dados *American Psychological Association, Cochrane Library*, EMBASE, *National Library of Medicine* e Scopus. Foram incluídas revisões sistemáticas que avaliaram diretrizes com recomendações para cuidados com a saúde mental de trabalhadores. Registro PROSPERO CRD42023461845.

**Resultados:**

identificaram-se quatro revisões sistemáticas publicadas entre 2015 e 2018. Os resumos evidenciaram ações facilitadoras e inibitórias das recomendações, bem como as três categorias de intervenção: prevenção primária - proteção do trabalhador; prevenção secundária - promover a saúde mental do trabalhador; e prevenção terciária - apoiar, acompanhar e reabilitar o trabalhador no retorno ao trabalho.

**Conclusões:**

as intervenções baseiam-se na prevenção, promoção e reconhecimento precoce, apoio e reabilitação aos problemas de saúde mental.

## INTRODUÇÃO

A saúde mental é determinada por uma complexa interação entre tensões e vulnerabilidades individuais, sociais e estruturais^(1)^. Dentro dessas interações, o trabalho pode ser um fator de proteção ou um gerador de danos à saúde mental^(2)^. Estima-se que 15% dos adultos em idade produtiva apresentem algum tipo de transtorno mental em determinado momento durante a vida laboral^(3)^.

A Organização Mundial da Saúde (OMS) alerta que o adoecimento mental está entre as principais causas de incapacidade no trabalho, recomendando ações efetivas para melhoria da saúde mental dos trabalhadores, cumprindo o seu direito a local de trabalho seguro e saudável^(3)^.

A proposta da Agenda Mundial para os Objetivos do Desenvolvimento Sustentável (ODS), determinada pela cúpula das Nações Unidas, tem objetivos e metas a serem cumpridos até o ano de 2030. No seu 8º objetivo, descreve a valorização do trabalho decente para o crescimento econômico, visando às características estruturais e funcionais de trabalho que não prejudiquem a saúde dos trabalhadores^(4)^.

Para o cuidado com a saúde mental, os gestores devem criar e manter um local de trabalho mentalmente saudável. Ações combinadas de saúde mental no trabalho podem ser utilizadas nesta abordagem, com princípios orientadores e ancorados em: proteger a saúde mental, reduzindo os fatores de risco relacionados ao trabalho; promover a saúde mental, desenvolvendo os aspectos positivos do trabalho e do trabalhador; e abordar os problemas de saúde mental entre trabalhadores^(5)^.

Essas ações devem ser realizadas tanto no ambiente ocupacional quanto diretamente com o trabalhador envolvido, e têm sido classificadas como estratégias integradas entre métodos de prevenção primária, secundária e terciária^(5-7)^.

A primária possibilita prevenir a exposição aos estressores de forma proativa, principalmente orientada para o local de trabalho e focada nos trabalhadores, antes que os mesmos apresentem algum sintoma relacionado à doença mental. A secundária é voltada para modificar a reação do trabalhador diante do problema apresentado em uma fase inicial, mas que ocasiona impactos na saúde psicológica. Já a terciária é reativa, na medida em que ocorrem os problemas de saúde mental, envolvendo o tratamento dos trabalhadores afetados e o apoio, a reabilitação e o retorno ao trabalho^(6,7)^.

Os locais de trabalho são heterogêneos, complexos e dinâmicos, e a realização de ações preventivas integradas é um desafio para todos os envolvidos^(6)^. Portanto, as estratégias institucionais com enfoque em um nível de prevenção podem ser adotadas, contudo a coordenação entre esses níveis é relevante, pois falhas na prevenção primária requerem esforços de prevenção secundária e/ou terciária^(7)^ que poderiam ser evitados.

Neste contexto, a enfermagem desempenha um papel crucial nas estratégias de promoção da saúde dos trabalhadores, identificando os riscos relacionados ao trabalho, ações e orientações sobre o estilo de vida, além de outras necessidades de saúde dos trabalhadores. A compreensão das especificidades e limitações do processo produtivo também possibilita ao enfermeiro ampliar suas ações junto aos trabalhadores, visando transformar as condições de trabalho e promover a saúde de forma eficaz^(8)^.

Para instituir medidas de prevenção no local de trabalho, podem ser utilizadas recomendações indicadas por diretrizes, que constituem declarações que trazem recomendações destinadas a guiar tomada de decisão em determinada área a partir de evidências científicas publicadas, principalmente, nas revisões sistemáticas (RS)^(9)^.

As RS são uma abordagem científica rigorosa para avaliar e resumir as evidências relacionadas a questões específicas, que podem auxiliar na tomada de decisão^(10)^. Observa-se um aumento exponencial na publicação de RS. Destaca-se que aproximadamente 22 novas RS são publicadas todos os dias^(11)^. Desse modo, resumos de RS surgiram como uma metodologia para agrupar, avaliar e sintetizar sistematicamente os resultados de várias RS denominada *umbrella review* ^(10)^.

Há um reconhecimento crescente sobre a importância das questões de saúde mental para o local de trabalho, todavia é necessário adotar ações e intervenções de saúde mental eficazes e baseadas em evidências científicas^(12)^. Apesar de o adoecimento mental ser influente na saúde do trabalhador, em busca preliminar realizada no *Joanna Briggs Institute Evidence Synthesis* e na *Cochrane Library*, não se encontraram revisões abrangentes que sintetizassem evidências sobre recomendações para a promoção da saúde mental de trabalhadores.

Diante do exposto e da complexidade da abordagem da saúde mental no ambiente de trabalho e do incipiente conhecimento dos trabalhadores e gestores sobre as recomendações que norteiam as instituições para formulação de políticas institucionais, este estudo teve como pergunta de pesquisa: quais as recomendações de diretrizes para a promoção da saúde mental no local de trabalho?

## OBJETIVOS

Sumarizar as recomendações de diretrizes para a promoção da saúde mental no local de trabalho.

## MÉTODOS

### Tipo de estudo

Este estudo consiste em *umbrella review,* que tem como foco principal sumarizar os resultados de pesquisas existentes relacionadas a um determinado tópico ou questão^(10)^. Também pode ser denominada como visão geral, revisão geral de RS ou revisão abrangente. Esta revisão foi desenvolvida de acordo com a metodologia do *Joanna Briggs Institute* (JBI) para *umbrella review* e recomendações de acordo com o protocolo *Preferred Reporting Items for Systematic reviews and Meta-Analyses* (PRISMA)^(10,13)^. O estudo foi registrado na base de dados *International Prospective Register of Systematic Reviews* (PROSPERO) - Protocolo CRD42023461845.

### Pergunta de revisão

Para a busca dos estudos e formulação da questão de pesquisa, utilizou-se a estratégia PICo, mnemônico para “População” (trabalhadores), “Fenômeno de Interesse” (recomendações de diretrizes para promoção da saúde mental) e “Contexto” (local de trabalho). Assim, estabeleceu-se a seguinte pergunta de pesquisa: quais as recomendações de diretrizes para a promoção da saúde mental de trabalhadores no local de trabalho?

### Critérios de inclusão

Foram incluídos estudos de RS, disponíveis na íntegra, que identificaram e avaliaram diretrizes para promoção da saúde mental no local de trabalho e que abordaram intervenções organizacionais, individuais ou integradas.

### Estratégia de pesquisa

A busca das RS ocorreu em janeiro de 2021, e a atualização, em julho de 2023, em cinco bases de dados, tais como *Cochrane Library* - Wiley, PubMed - *National Library of Medicine, Scopus* - Elsevier, EMBASE - Elsevier e *American Psychological Association* (PsycINFO) - EBSCO.

Para a estratégia de busca, utilizaram-se os descritores controlados “*mental health*”, “*workplace*”, “*guideline*” e “*systematic review*”, associados ao operador booleano “*AND*”, com adequação aos descritores próprios de cada base de dados. Segundo recomendação do JBI *Manual for Evidence Synthesis* nos descritores, incluiu-se “*systematic review*”, considerando que algumas bases de dados não possuem um filtro de busca pré-definido para artigos de revisão^(10)^. Não houve delimitação de idioma ou temporalidade.

### Seleção dos estudos

O processo de identificação e seleção dos estudos de RS ocorreu em quatro etapas, seguindo a recomendação de acordo com os itens do PRISMA. Na primeira, foi realizada a identificação dos registros nas bases de dados; na segunda, ocorreu a seleção dos registros rastreados por meio da leitura dos títulos e resumos; na terceira, a elegibilidade dos registros foi avaliada de acordo com a pergunta de pesquisa; e na quarta, foram incluídos os estudos de RS, conforme apresentado na Figura 1. Além disso, a lista de referências das RS identificadas e incluídas também foi examinada para identificar quaisquer outras RS não encontradas na busca inicial das bases de dados eletrônicas^(13)^.

**Figura 1:** Etapas da identificação e seleção de revisões sistemáticas sobre recomendações de diretrizes para a promoção da saúde mental no local de trabalho, Londrina, Paraná, Brasil, 2023

Para redução do viés de pesquisa, os registros foram examinados por dois revisores independentes, após a inspeção de títulos e resumos, bem como da leitura do texto completo dos artigos. As divergências foram resolvidas por consenso e, em caso de dúvidas, um terceiro revisor foi consultado. Para organização e gerenciamento dos dados, utilizou-se um instrumento elaborado no *software Microsoft Excel^®^*, versão 2021, que contemplava as informações referentes ao título dos estudos, ano de publicação, se responde à pergunta de pesquisa e motivo de exclusão do estudo. Neste *software*, também foi realizada uma triagem detalhada dos os estudos, e as duplicatas foram removidas.

### Avaliação da qualidade das revisões sistemáticas

A fim de examinar a qualidade das RS incluídas, utilizou-se a lista de verificação de avaliação crítica do JBI para RS e sínteses de pesquisas. A lista contempla 11 itens com opções de resposta “sim”, “não”, “não está claro” e “não aplicável”^(10)^. Os dados foram extraídos por um pesquisador e verificados por um segundo pesquisador. As discrepâncias foram resolvidas por consenso.

### Extração dos dados das revisões sistemáticas

Utilizou-se uma versão personalizada da ferramenta de extração de dados do JBI *Manual for Evidence Synthesis* ^(10)^. Assim, as seguintes características foram coletadas: 1) autor/ano; 2) objetivos da RS incluída; 3) ambiente e contexto; 4) número de bancos de dados pesquisados; 5) número de diretrizes incluídas em cada RS; 6) intervalo de datas de publicação das diretrizes incluídas na RS; 7) instrumento utilizado para avaliar a qualidade das diretrizes; e 8) principais resultados relatados.

### Síntese das revisões sistemáticas incluídas

Os resultados de interesse extraídos das RS incluídas são apresentados de forma narrativa. Foram incluídas figuras, quadros e tabelas, quando apropriadas, para auxiliar na apresentação dos dados. Para exposição do resumo das evidências, utilizou-se uma indicação visual, na qual são destacadas em verde as ações facilitadoras e, em vermelho, as ações inibitórias, para promoção da saúde mental no local de trabalho, conforme recomendação do JBI *Manual for Evidence Synthesis* ^(10)^.

As recomendações para a promoção da saúde mental no local de trabalho envolvem três níveis de prevenção. Neste estudo, denominaram-se recomendações de prevenção primária as intervenções que visam prevenir problemas de saúde mental relacionados ao trabalho, com o objetivo de reduzir os estressores do trabalho, ou relacionadas às estruturas do local de trabalho^(14)^.

Como recomendações de prevenção secundária, foram consideradas aquelas que se direcionam aos trabalhadores, com o objetivo de melhorar a capacidade deles para enfrentar e resistir aos estressores do local de trabalho, além de impedir o progresso de problemas de saúde mental subclínicos para transtornos diagnosticáveis^(14)^. E definiu-se prevenção terciária como aquela que envolve o tratamento de trabalhadores com acometimento de saúde mental e o apoio e a reabilitação para o trabalho^(14)^.

## RESULTADOS

Inicialmente, esta pesquisa identificou 277 artigos e, após a seleção dos estudos, foram selecionadas quatro RS^(15-18)^, constituindo esta *umbrella review*. De forma complementar, realizou-se a leitura da lista de referências das RS incluídas, contudo nenhum estudo foi selecionado, conforme apresentado na Figura 1.

A Tabela 1 apresenta a qualidade geral das RS incluídas, com todas obtendo pontuação de 100%, nos itens 2-5 e 11, de 75%, nos itens 6-8 e 10, e o item 1 obteve 50% de adequação. Já o item 9, que avalia o risco de viés, não foi atendido em nenhum dos estudos.

**Tabela 1:** Avaliação da qualidade das revisões sistemáticas incluídas usando a ferramenta *Joanna Briggs Institute*, Londrina, Paraná, Brasil, 2023

Itens da JBI	Autor/ano				Porcentagem (%) dos critérios atendidos
	Joosen *et al*., 2015^(15)^	Dewa *et al.*, 2016^(16)^	Memish *et al.*, 2017^(17)^	Nexo *et al.*, 2018^(18)^	
1	S	NC	S	NC	50
2	S	S	S	S	100
3	S	S	S	S	100
4	S	S	S	S	100
5	S	S	S	S	100
6	NC	S	S	S	75
7	NC	S	S	S	75
8	S	S	NC	S	75
9	N	N	N	N	0
10	S	S	NC	S	75
11	S	S	S	S	100

*S - Sim; N - Não; NC - Não está claro; NA - Não aplicável;*

*Itens da ferramenta JBI: 1. A pergunta da revisão está clara e explicitamente declarada? 2. Os critérios de inclusão foram adequados para a pergunta de revisão? 3. A estratégia de busca foi adequada? 4. As fontes e recursos utilizados para a busca dos estudos foram adequados? 5. Os critérios de avaliação dos estudos foram adequados? 6. A avaliação crítica foi conduzida por dois ou mais revisores independentemente? 7. Havia métodos para minimizar erros na extração de dados? 8. Os métodos usados para combinar estudos foram apropriados? 9. A probabilidade de viés de publicação foi avaliada? 10. As recomendações para políticas e/ou práticas foram apoiadas pelos dados relatados? 11. As diretrizes específicas para novas pesquisas foram adequadas?*

Os autores das RS selecionadas realizaram as buscas entre quatro e 11 locais de busca. As diretrizes foram encontradas em repositórios de organizações de segurança e saúde ocupacional, órgãos governamentais^(16,17)^, bancos de dados de diretrizes^(15,16,18)^ e a ferramenta *Google^®^* ^(15-18)^ e bases de dados como PubMed, PsycNET, *Web of Science* e *Arts & Humanities Citation Index* ^(15,18)^.

As diretrizes foram desenvolvidas para locais de trabalho, empregadores e empregados, e elaboradas do ano de 2003 a 2015. Quanto ao quantitativo de diretrizes incluídas nas RS, variaram de três a 20 diretrizes, totalizando 54 diretrizes. Para avaliação da qualidade das diretrizes, as revisões utilizaram o *Guidelines for Research and Evaluation* II (AGREE II).

No Quadro 1, são apresentadas as características das RS que avaliaram diretrizes para promoção da saúde mental no local de trabalho em relação ao autor, ano de publicação, objetivos, quantitativo de diretrizes incluídas, avaliação AGREE II e país ou organização de desenvolvimento das diretrizes.

**Quadro 1:** Características das revisões sistemáticas que avaliaram diretrizes para promoção da saúde mental no local de trabalho em relação ao autor, ano de publicação, objetivos, quantitativo de diretrizes incluídas e país ou organização de desenvolvimento das diretrizes, Londrina, Paraná, Brasil, 2023

Autor/ano	Objetivos	Diretrizes incluídas	AGREE II^*^	País ou organização de desenvolvimento das diretrizes
Joosen *et al.*, 2015^(15)^	Identificar diretrizes de saúde ocupacional com foco no manejo de transtornos mentais e sintomas relacionados ao estresse, de diferentes países do mundo, e descrevê-las, comparar o conteúdo e avaliar sua qualidade de desenvolvimento e relato.	14 diretrizes	Média AGREE II 61,5%	Holanda, Reino Unido, Japão, Finlândia e República da Coreia
Dewa *et al.*, 2016^(16)^	Identificar diretrizes de melhores práticas para incapacidade relacionadas a doenças mentais para empregadores na literatura cinza.	Três diretrizes	Média AGREE II 72,2%	Canadá, Reino Unido e Austrália
Memish *et al.*, 2017^(17)^	Determinar a qualidade das diretrizes de saúde mental no local de trabalho; avaliar a abrangência das recomendações abordando os três segmentos da abordagem integrada: prevenção de danos e minimizar os fatores de risco no ambiente de trabalho; promovendo fatores positivos e de proteção no local de trabalho; e gerenciamento de problemas de saúde mental, independentemente da causa.	20 diretrizes	Média AGREE II 59,8%	Canadá, Reino Unido, Austrália, União Europeia, Organização Mundial da Saúde
Nexo *et al.*, 2018^(18)^	Revisar sistematicamente a qualidade de desenvolvimento das diretrizes que fornecem recomendações para os atores no local de trabalho para prevenir adoecimento mental relacionado ao trabalho, detectar e gerenciar sinais precoces ou facilitar o retorno ao trabalho; e comparar o conteúdo das intervenções recomendadas incluídas nas diretrizes e as evidências relatadas que sustentam os resultados esperados das intervenções.	17 diretrizes	Média AGREE II 54,5%	Austrália, Canadá, Dinamarca, Inglaterra, Nova Zelândia, Suécia, Organização para Cooperação Desenvolvimento Econômico, Organização Mundial da Saúde

* *Porcentagem de adequação segundo Guidelines for Research and Evaluation II.*

O resumo das evidências com base nas características apresentadas nas RS é apresentado no Quadro 2. Na descoberta sintetizada, são destacadas em verde as ações facilitadoras e, em vermelho, as ações inibitórias, para promoção da saúde mental no local de trabalho.

**Quadro 2:** Resumo das recomendações apresentadas nas revisões sistemáticas para promoção da saúde mental no local de trabalho, Londrina, Paraná, Brasil, 2023

Fenômenos de interesse	Autores	Descoberta sintetizada
Manejo dos transtornos mentais e sintomas relacionados ao estresse.	Joosen *et al.*, 2015^(15)^	Avaliação dos fatores do local de trabalho relevantes para a saúde mental. Avaliação das competências e habilidades no trabalho. Avaliação da carga de trabalho, estressores e conteúdo do trabalho. Avaliação das habilidades de comunicação e/ou resolução de problemas entre trabalhador e supervisor. Avaliação dos sintomas da saúde mental dos trabalhadores. Examinar fatores que influenciam a recuperação na vida privada e profissional. Recomendações sobre estratégias de enfrentamento (habilidades de comunicação e de resolução de problemas). Avaliação do risco de autodestruição/suicídio. Aconselhamento com início precoce, orientação e apoio. Tratamento específico de saúde mental intervenções psicológicas, intervenções cognitivas e comportamentais, estratégias de autogestão, intervenções de retorno ao trabalho, adaptações no trabalho com redução das condições estressantes e/ou redução da jornada e demanda no trabalho, proibição dos plantões noturnos, aconselhamento ao empregador para manter o contato com o trabalhador e dar instruções aos colegas de trabalho para evitar o estigma. Acompanhamento do trabalhador pelos líderes e outros profissionais envolvidos no processo de recuperação e avaliação da capacidade para o trabalho.
		Tratamento medicamentoso indicado somente em transtornos mentais graves ou insônia.
Melhores práticas para incapacidade relacionadas a doenças mentais para empregadores.	Dewa *et al.*, 2016^(16)^	Desenvolvimento de políticas/procedimentos organizacionais. Instituição de um ambiente de trabalho favorável. Instituição de plano para a licença por invalidez, com comunicação regular entre a organização e o trabalhador. Retorno ao trabalho coordenado, planejado e facilitado por um coordenador designado que apoie a comunicação entre o líder, o trabalhador e a organização. Oferta de intervenções intensivas, multidisciplinares e baseadas em evidências. Apoio aos trabalhadores para obter os tratamentos disponíveis. Recomendação do treinamento dos líderes. Fornecimento de treinamento e conscientização em saúde mental para todos os trabalhadores para evitar o estigma. Avaliação do trabalho realizado pelo trabalhador e pelo supervisor.
		Intervenções clínicas sem modificações no trabalho e/ou orientação profissional.
Recomendações abordando os três segmentos da abordagem integrada no gerenciamento de problemas de saúde mental.	Memish *et al.*, 2017^(17)^	Recomendações para a organização minimizar os fatores de risco e promover fatores positivos no trabalho. Recomendações para prevenção do adoecimento mental direcionadas ao nível individual. Desenvolvimento de estilos de liderança, clima ou cultura organizacional positivos no local de trabalho. Detecção e tratamento dos problemas de saúde mental no local de trabalho.
		Falta de recursos e/ou pessoal capacitado para implementação das intervenções.
Recomendações para prevenir, detectar e gerenciar sinais precoce de adoecimento mental relacionado ao trabalho ou facilitar o retorno ao trabalho.	Nexo *et al.*, 2018^(18)^	Política de saúde mental em todos os níveis da organização (envolvimento dos trabalhadores, planejamento, recursos, funções e responsabilidades). Identificação e eliminação contínua e sistemática dos riscos psicossociais. Melhora da concepção do trabalho e adequação da pessoa-trabalho por meio de treinamento de habilidades e horários de trabalho rotativos em locais onde os riscos psicossociais não podem ser eliminados. Implementação de programas educacionais para conscientizar sobre a saúde mental e evitar o estigma associado ao adoecimento mental. Estimulo de fatores positivos do trabalho, como o apoio dos colegas e recompensas. Aumento da comunicação e competências relacionais dos líderes para evitar adoecimento mental. Avaliação da saúde mental dos trabalhadores. Estratégias para gerenciamento do estresse e dos problemas de saúde mental. Realização de aconselhamento no trabalho e adequações para trabalhadores com adoecimento mental. Estimulo de apoio/orientação entre os colegas de trabalho. Abordagem de retorno ao trabalho coordenada e multidisciplinar. Treinamento dos líderes para detectar sinais de adoecimento mental.
		Gestão inadequada do clima de trabalho, como assédio moral e conflitos no trabalho. Trabalho mal organizado com altas demandas, baixo controle e ameaças de violência por parte dos clientes.

As RS apresentaram as recomendações de diretrizes para a saúde mental no local de trabalho em nível primário^(15,17,18)^, em nível secundário^(15,17,18)^ e em nível terciário^(15-18)^. Destacou-se, na avaliação das diretrizes, o predomínio de recomendações integradas entre os níveis de prevenção (74%).

A Figura 2 traz a síntese das principais recomendações apresentadas nas RS agrupadas, de acordo com o nível de prevenção, sendo: 1) Recomendações de prevenção primária - proteção do trabalhador; 2) Recomendações de prevenção secundária - detectar e promover a saúde mental do trabalhador; e 3) Recomendações de prevenção terciária - apoiar, acompanhar e reabilitar o trabalhador no retorno ao trabalho após afastamento. A capacitação dos líderes foi recomendada para os três níveis de prevenção.

**Figura 2:** Síntese dos níveis de prevenção das principais recomendações identificadas nas revisões sistemáticas que avaliaram as diretrizes para promoção da saúde mental no local de trabalho, Londrina, Paraná, Brasil, 2023

## DISCUSSÃO

Observou-se que as recomendações das diretrizes identificadas neste estudo para promoção da saúde mental no local de trabalho se baseiam na proteção e promoção da saúde mental, reconhecimento precoce dos problemas, apoio e reabilitação do trabalhador no retorno ao trabalho.

Na abordagem da saúde mental, é importante entender que cada organização tem suas necessidades, e isso exigirá um conjunto de estratégias a serem definidas e abordadas por um programa de saúde integral. Desse modo, é necessário estimular a cooperação e participação de empregadores, líderes e trabalhadores, de forma a responsabilizar e mobilizar os conhecimentos necessários para obter melhores resultados em saúde mental^(19)^.

No que concerne à avaliação das diretrizes, o AGREE II, utilizado nas revisões, oferece oportunidade de avaliar, de forma sistemática, específica e objetiva, a qualidade das diretrizes elencadas nos estudos analisados. Contudo, embora esse instrumento seja utilizado para avaliar o rigor e a transparência das diretrizes, ele não estabelece distinção entre alta e baixa qualidade. Também é destacada a subjetividade das avaliações, podendo os avaliadores interpretar os itens e a pontuação de formas diferentes^(9)^.

Em consonância com os resultados encontrados, durante a concepção deste estudo, a OMS publicou a diretriz para saúde mental no trabalho que corroborou os achados desta pesquisa. Na diretriz da OMS, é destacado que a abordagem da saúde mental deve ser ampla e abrangente, e que é importante que os ambientes de trabalho adotem um cenário propício para as mudanças, com comprometimento para combater o estigma e a discriminação, coordenando abordagens multissetoriais e participativas para intervenções eficazes^(20)^.

Para o desenvolvimento de um ambiente de trabalho saudável, é fundamental a participação compartilhada de empregadores, gestores e trabalhadores. Neste contexto, é imperativo fornecer informações sobre como reduzir os riscos que potencialmente causem danos e como potencializar os fatores no local de trabalho que promovam a saúde psicológica, constituindo-se na prevenção primária dos agravos em saúde mental^(21)^.

A avaliação do estado de saúde mental e bem-estar dos trabalhadores é um ponto relevante para mensurar as necessidades da organização. Isso pode incluir uma avaliação dos indicadores de saúde mental e bem-estar, nível de conhecimento em saúde mental e identificação de fontes de estresse no local de trabalho. A partir de uma avaliação criteriosa do local de trabalho, poderão ser implementadas iniciativas específicas para minimizar os riscos e/ou as necessidades da organização, a fim de evitar ou reduzir os agravos na saúde mental dos trabalhadores^(22)^.

Um ambiente de trabalho que prioriza ações para proteção à saúde mental deve mitigar os fatores de agravos relacionados ao trabalho, com identificação, controle e eliminação dos riscos que possam ser eliminados e avaliação e controle dos riscos que não possam ser eliminados. É necessário instituir políticas que reduzam a tensão no local de trabalho, construindo um clima organizacional positivo^(21)^.

A política institucional deve estar alicerçada no respeito, consideração e tolerância, reconhecendo a diversidade, abordando com severidade o comportamento desrespeitoso, a violência, a discriminação, o assédio e a incivilidade, contribuindo, desse modo, para que a cultura organizacional represente os valores compartilhados da instituição^(22)^.

A configuração do trabalho é reconhecida como um dos fatores de risco que ocasionam impacto psicológico nos trabalhadores. Assim, é necessário analisar a maneira como as tarefas são organizadas, garantir que a carga de trabalho esteja alinhada com as capacidades e recursos do trabalhador, equilibrar as exigências com o controle que os trabalhadores têm sobre seu trabalho, oferecer oportunidades de desenvolvimento de carreira, melhorar a comunicação e, se possível, flexibilizar a escala de trabalho^(22)^. Outras ações incluem garantir a harmonia entre trabalho e vida, fornecer mais autonomia sobre como o trabalho é realizado, respeitar os limites entre o horário de trabalho e folga, envolvê-los nas decisões, construir uma cultura de reconhecimento e fornecer salário digno^(23)^.

A designação das atividades do trabalhador também deve ser considerada, sendo indicado combinar as competências psicológicas de um trabalhador com as demandas do trabalho. Outra estratégia que o ambiente ocupacional pode oferecer aos indivíduos, com o intuito de atrair, reter e desenvolver trabalhadores, é proporcionar oportunidades para crescimento e desenvolvimento para o aprimoramento de suas habilidades, além de garantir que obtenham *feedback* relevante do seu desempenho^(21,23)^. As intervenções que envolvem as abordagens participativas podem ser consideradas para obter melhores resultados na redução do sofrimento emocional^(20)^.

Em relação às recomendações para a prevenção secundária, a promoção da saúde dos trabalhadores pode ser integrada às iniciativas de prevenção. Além das ações executadas pela organização, é necessário que os trabalhadores também assumam a responsabilidade de cuidar da sua saúde mental, associando à promoção das abordagens preventivas e multiplicando os benefícios^(7)^.

Para que os trabalhadores compreendam a importância das medidas de prevenção, são necessários o conhecimento e a conscientização em saúde mental. Isso requer informações básicas, explicitando o que é um problema de saúde mental, os sinais e sintomas e problemas comuns de saúde mental, o que fazer caso precise de apoio ou tenha algum colega necessitando de ajuda^(7)^. A compreensão sobre a saúde mental facilita o reconhecimento, a identificação e o gerenciamento de problemas psicológicos, ajudando a reduzir o estigma e aumentar a conscientização sobre as opções de autocuidado^(7,20)^.

Entre as estratégias para estabelecer comportamentos de autocuidado, estão a alimentação saudável, dormir bem, participar de atividades relaxantes, engajamento com familiares e amigos, desenvolvimento de hábitos saudáveis, não fumar ou abusar do álcool, cultivar relacionamentos saudáveis e praticar atividade física^(7,24)^.

Para que ocorra a identificação dos problemas de saúde mental, é necessária a investigação^(7,20)^, que pode ser feita por meio de questionários autorrelatados. A relevância desta estratégia baseia-se na identificação e abordagem dos problemas de saúde psicológica quando estão em estágio inicial^(25)^. Nas recomendações, não há evidência de benefícios ou danos potenciais nas intervenções de triagem dos trabalhadores^(20)^. Desse modo, realizar o rastreamento da situação de saúde mental, além de permitir a autoavaliação do trabalhador, pode contribuir para o planejamento institucional.

As intervenções individuais podem ser utilizadas na prevenção e no gerenciamento do sofrimento psicológico, limitando o surgimento de problemas mais graves de saúde mental. As intervenções podem ser desenvolvidas presencialmente ou digitalmente, por meio de aplicativos ou *online* ^(20)^.

As estratégias se concentram em habilidades interpessoais, gerenciamento do estresse, resolução de conflitos, resolução de problemas, comunicação, gerenciamento de tempo, atenção plena, resiliência, habilidades positivas de enfrentamento, técnicas de relaxamento, atividade física e intervenções no estilo de vida^(7,20,24)^. Também são utilizadas intervenções psicológicas baseadas na terapia de comportamento cognitivo, terapia de aceitação e compromisso e de psicologia positiva^(20)^.

A promoção da saúde mental no trabalho é uma tarefa complexa e envolve fortalecer capacidades, conscientizar e oferecer oportunidades para reconhecer e agir precocemente sobre as condições de saúde mental^(20)^.

Nas recomendações para prevenção terciária, em caso de doença mental estabelecida, podem-se realizar ações com o objetivo de reduzir o sofrimento e a disfunção associada a um distúrbio mental. As ações podem envolver a garantia de acesso imediato ao tratamento apropriado, proporcionando melhora na qualidade de vida e engajamento no trabalho, contribuindo para a recuperação dos problemas de saúde mental^(26)^.

No gerenciamento de problemas de saúde mental, a intervenção precoce é fundamental. Para tanto, devem-se divulgar os serviços de apoio disponíveis que os trabalhadores podem procurar ajuda, além de explicitar os benefícios do auxílio profissional^(27)^. As intervenções individuais também podem ser consideradas para reduzir os sintomas e melhorar a eficácia no trabalho.

Em casos em que haja a necessidade de afastamento das atividades laborais, são necessárias intervenções para reabilitação e retorno ao trabalho^(20)^. Estudo de metanálise que avaliou essas intervenções identificou que as estratégias se baseiam nos mecanismos de enfrentamento, gerenciamento do estresse, técnicas de relaxamento, compartilhamento de experiências pessoais com fatores estressantes relacionados ao trabalho. Também são realizadas intervenções de coordenação com gerenciamento de caso, educação e treinamento, avaliação das cargas de trabalho, melhora da comunicação e aumento da acessibilidade entre o trabalhador e seu supervisor. Ainda é relatado que a gestão de caso, composta por equipes multidisciplinares e a adoção de estratégias combinadas, é mais eficaz^(28)^.

As iniciativas de retorno ao trabalho devem visar à adaptação dos ambientes de trabalho para corresponder às capacidades, necessidades e preferências do trabalhador, devendo ser personalizadas para cada trabalhador. O apoio à reabilitação e retorno ao trabalho propicia maiores possibilidades de alcançar resultados positivos, com chances reduzidas de recorrência de transtornos mentais^(20)^.

Além das formas citadas, tem-se as recomendações integradas que abrangem a coordenação entre os três níveis de prevenção, possibilitando realizar ações de prevenção de forma sinérgica^(7)^. As organizações devem normalizar e apoiar a saúde mental, considerando a saúde mental e o bem-estar como prioridades, oferecendo serviços de apoio e prevenção. Líderes e gerentes em toda a organização devem ser apoiados para criar uma cultura de cuidados com a saúde mental^(23)^.

A liderança exerce papel fundamental em todos os níveis de prevenção, seja no desenvolvimento de um ambiente de trabalho mentalmente saudável ou no reconhecimento de funcionários com sintomas psicológicos, atuando de forma transversal para melhorar a saúde mental^(20,29)^.

Para tanto, gerentes e supervisores, ou seja, líderes, necessitam de capacitação para realizar a detecção e resposta precoce e apoio para o retorno ao trabalho. A capacitação pode envolver o desenvolvimento de habilidades básicas no gerenciamento de pessoas e do fluxo de trabalho e habilidades de delegação, que irão contribuir para prevenção do adoecimento mental, como abordar e conversar com um trabalhador com uma condição de saúde mental e como encaminhá-los para os recursos de ajuda disponíveis na organização^(29)^.

Além disso, os líderes devem empenhar-se para reduzir o estigma associado aos problemas de saúde mental e promover uma cultura de trabalho com transparência, confiança, respeito, abertura, igualdade, inclusão, empatia e apoio, ao invés de comparar e promover um ambiente hostil de competição^(24)^.

Para detecção e resposta precoce, é importante que os líderes conheçam as personalidades e comportamentos dos trabalhadores, no intuito de identificar precocemente caso haja alguma alteração relativamente ao comportamento habitual^(29)^. Outrossim, o desenvolvimento da competência dos líderes na saúde mental é fundamental e pode melhorar o conhecimento, as atitudes e o comportamento de busca por ajuda dos trabalhadores^(20)^.

Nas intervenções, também são identificados líderes que não priorizam a estratégia institucional, fazem cobranças aos trabalhadores, atuam dificultando a implementação e a transição do aprendizado para o local de trabalho. Do mesmo modo, o relacionamento prejudicado com os trabalhadores também é evidenciado como uma barreira para a abrangência e sustentabilidade das intervenções^(27)^.

As evidências científicas recomendam a capacitação de líderes para apoiar a saúde mental^(20)^, visto que a influência da liderança é tão relevante, já que podem bloquear ou impedir a implementação de mudanças ou minar a eficácia de quaisquer mudanças realizadas^(29)^. Desse modo, a sensibilização, o estigma e estereótipos relacionados ao adoecimento mental devem ser abordados junto aos líderes no intuito de reduzir as barreiras para implementação das intervenções^(27)^.

Alguns fatores também podem interferir no processo de intervenção, destacando-se a falta de treinamento adequado dos envolvidos, o tempo insuficiente durante o dia de trabalho para os participantes, as prioridades conflitantes, os problemas de carga de trabalho e a falta da cobertura dos recursos humanos^(27)^.

Estudo de RS, que buscou identificar quais os fatores críticos e de sucesso para a implementação de práticas de saúde e bem-estar no trabalho, identificou como fator de sucesso a comunicação regular entre as estruturas de aprendizado e de governança que possa propiciar adaptações locais durante a implementação da estratégia na organização. Como fatores críticos, são destacados a falta de continuidade do esforço e de adaptação das intervenções, o nível de envolvimento, o apoio e o compromisso organizacional com a intervenção^(29)^.

A implementação de estratégias para um local de trabalho psicologicamente saudável se destaca por ser complexa. Portanto, uma etapa fundamental é o processo de avaliação, que pode monitorar, revisar e identificar oportunidades de melhorias específicas para cada realidade institucional^(5)^.

As RS destacaram que diretrizes com recomendações para a saúde mental no local de trabalho são difíceis de detectar, sendo identificadas, principalmente, através da literatura cinza. Outro ponto relevante é que o conteúdo e a qualidade das diretrizes variaram significativamente de acordo com a pontuação AGREE II, o que pode ter ocorrido em decorrência da subjetividade da avaliação, bem como da falta de atender algum critério no momento de avaliação^(17,18)^.

### Limitações do estudo

Como limitações do presente estudo, destaca-se que a busca foi limitada às bases de dados científicas, não incluindo fontes da literatura cinza. Além disso, cabe salientar o quantitativo reduzido de RS encontradas, bem como a carência de RS publicadas nos últimos anos. Outrossim, as RS e diretrizes avaliadas foram elaboradas em países desenvolvidos. Esse achado demonstra a necessidade de ampliar a investigação e elaboração de recomendações de qualidade para promoção da saúde mental no local de trabalho em países subdesenvolvidos. Todavia, acredita-se que esse achado não compromete a qualidade da pesquisa, mas indica a relevância de estudos futuros para o desenvolvimento de recomendações e avaliação da saúde do trabalhador com a implementação das recomendações de diretrizes para promoção da saúde mental.

### Contribuições para as áreas da enfermagem e saúde

A sumarização das recomendações descritas nas diretrizes avaliadas em relação à qualidade da formulação pelas RS contribui para disseminação do conhecimento, e pode colaborar para que os gestores e enfermeiros do trabalho implementem estratégias personalizadas de acordo com a sua realidade, cumprindo o papel de potencializar a promoção da saúde mental.

## CONCLUSÕES

Os resultados desta *umbrella review* apresentaram uma síntese das recomendações de diretrizes para a promoção da saúde mental no local de trabalho. As intervenções baseiam-se na prevenção, promoção e reconhecimento precoce, apoio e reabilitação quanto aos problemas de saúde mental. Ressalta-se que as diretrizes se constituem ferramentas valiosas para sintetizar e traduzir as evidências científicas em recomendações, que podem e devem ser utilizadas por gestores no local de trabalho.

A partir do pressuposto que o adoecimento mental entre os trabalhadores é um crescente problema de saúde pública, o local de trabalho deve constituir-se um ambiente que garanta ações para prevenir, promover, detectar e gerenciar problemas de saúde mental.
