# Supplementary material for: ERRATUM
Source: Rev Bras Enferm. 2025 Mar 14;78(1):e2025n1e03. doi: 10.1590/0034-7167.20257801e03 (PMC11913042; doi:10.1590/0034-7167.20257801e03)
Supplement: Related texts

# Correction: ERRATA

**DOI:** 10.1590/0034-7167.20257801e03pt

No artigo “Recomendações de diretrizes para promoção da saúde mental no local de trabalho: umbrella review”, com número DOI: https://doi.org/10.1590/0034-7167-2024-0086pt, publicado no periódico Revista Brasileira de Enfermagem, 2024;77(6):e20240086, na página 5:

Onde se lia:

Substitui-se o Quadro 2:

**Quadro 2:** Resumo das recomendações apresentadas nas revisões sistemáticas para promoção da saúde mental no local de trabalho, Londrina, Paraná, Brasil, 2023

Fenômenos de interesse	Autores	Descoberta sintetizada
Manejo dos transtornos mentais e sintomas relacionados ao estresse.	Joosen *et al.*, 2015^(15)^	Avaliação dos fatores do local de trabalho relevantes para a saúde mental. Avaliação das competências e habilidades no trabalho. Avaliação da carga de trabalho, estressores e conteúdo do trabalho. Avaliação das habilidades de comunicação e/ou resolução de problemas entre trabalhador e supervisor. Avaliação dos sintomas da saúde mental dos trabalhadores. Examinar fatores que influenciam a recuperação na vida privada e profissional. Recomendações sobre estratégias de enfrentamento (habilidades de comunicação e de resolução de problemas). Avaliação do risco de autodestruição/suicídio. Aconselhamento com início precoce, orientação e apoio. Tratamento específico de saúde mental intervenções psicológicas, intervenções cognitivas e comportamentais, estratégias de autogestão, intervenções de retorno ao trabalho, adaptações no trabalho com redução das condições estressantes e/ou redução da jornada e demanda no trabalho, proibição dos plantões noturnos, aconselhamento ao empregador para manter o contato com o trabalhador e dar instruções aos colegas de trabalho para evitar o estigma. Acompanhamento do trabalhador pelos líderes e outros profissionais envolvidos no processo de recuperação e avaliação da capacidade para o trabalho.
		Tratamento medicamentoso indicado somente em transtornos mentais graves ou insônia.
Melhores práticas para incapacidade relacionadas a doenças mentais para empregadores.	Dewa *et al.*, 2016^(16)^	Desenvolvimento de políticas/procedimentos organizacionais. Instituição de um ambiente de trabalho favorável. Instituição de plano para a licença por invalidez, com comunicação regular entre a organização e o trabalhador. Retorno ao trabalho coordenado, planejado e facilitado por um coordenador designado que apoie a comunicação entre o líder, o trabalhador e a organização. Oferta de intervenções intensivas, multidisciplinares e baseadas em evidências. Apoio aos trabalhadores para obter os tratamentos disponíveis. Recomendação do treinamento dos líderes. Fornecimento de treinamento e conscientização em saúde mental para todos os trabalhadores para evitar o estigma. Avaliação do trabalho realizado pelo trabalhador e pelo supervisor.
		Intervenções clínicas sem modificações no trabalho e/ou orientação profissional.
Recomendações abordando os três segmentos da abordagem integrada no gerenciamento de problemas de saúde mental.	Memish *et al.*, 2017^(17)^	Recomendações para a organização minimizar os fatores de risco e promover fatores positivos no trabalho. Recomendações para prevenção do adoecimento mental direcionadas ao nível individual. Desenvolvimento de estilos de liderança, clima ou cultura organizacional positivos no local de trabalho. Detecção e tratamento dos problemas de saúde mental no local de trabalho.
		Falta de recursos e/ou pessoal capacitado para implementação das intervenções.
Recomendações para prevenir, detectar e gerenciar sinais precoce de adoecimento mental relacionado ao trabalho ou facilitar o retorno ao trabalho.	Nexo *et al.*, 2018^(18)^	Política de saúde mental em todos os níveis da organização (envolvimento dos trabalhadores, planejamento, recursos, funções e responsabilidades). Identificação e eliminação contínua e sistemática dos riscos psicossociais. Melhora da concepção do trabalho e adequação da pessoa-trabalho por meio de treinamento de habilidades e horários de trabalho rotativos em locais onde os riscos psicossociais não podem ser eliminados. Implementação de programas educacionais para conscientizar sobre a saúde mental e evitar o estigma associado ao adoecimento mental. Estimulo de fatores positivos do trabalho, como o apoio dos colegas e recompensas. Aumento da comunicação e competências relacionais dos líderes para evitar adoecimento mental. Avaliação da saúde mental dos trabalhadores. Estratégias para gerenciamento do estresse e dos problemas de saúde mental. Realização de aconselhamento no trabalho e adequações para trabalhadores com adoecimento mental. Estimulo de apoio/orientação entre os colegas de trabalho. Abordagem de retorno ao trabalho coordenada e multidisciplinar. Treinamento dos líderes para detectar sinais de adoecimento mental.
		Gestão inadequada do clima de trabalho, como assédio moral e conflitos no trabalho. Trabalho mal organizado com altas demandas, baixo controle e ameaças de violência por parte dos clientes.

Pela versão em cores do Quadro 2:

**Quadro 2:** Resumo das recomendações apresentadas nas revisões sistemáticas para promoção da saúde mental no local de trabalho, Londrina, Paraná, Brasil, 2023

Fenômenos de interesse	Autores	Descoberta sintetizada
Manejo dos transtornos mentais e sintomas relacionados ao estresse.	Joosen *et al.*, 2015^(15)^	Avaliação dos fatores do local de trabalho relevantes para a saúde mental. Avaliação das competências e habilidades no trabalho. Avaliação da carga de trabalho, estressores e conteúdo do trabalho. Avaliação das habilidades de comunicação e/ou resolução de problemas entre trabalhador e supervisor. Avaliação dos sintomas da saúde mental dos trabalhadores. Examinar fatores que influenciam a recuperação na vida privada e profissional. Recomendações sobre estratégias de enfrentamento (habilidades de comunicação e de resolução de problemas). Avaliação do risco de autodestruição/suicídio. Aconselhamento com início precoce, orientação e apoio. Tratamento específico de saúde mental intervenções psicológicas, intervenções cognitivas e comportamentais, estratégias de autogestão, intervenções de retorno ao trabalho, adaptações no trabalho com redução das condições estressantes e/ou redução da jornada e demanda no trabalho, proibição dos plantões noturnos, aconselhamento ao empregador para manter o contato com o trabalhador e dar instruções aos colegas de trabalho para evitar o estigma. Acompanhamento do trabalhador pelos líderes e outros profissionais envolvidos no processo de recuperação e avaliação da capacidade para o trabalho.
		Tratamento medicamentoso indicado somente em transtornos mentais graves ou insônia.
Melhores práticas para incapacidade relacionadas a doenças mentais para empregadores.	Dewa *et al.*, 2016^(16)^	Desenvolvimento de políticas/procedimentos organizacionais. Instituição de um ambiente de trabalho favorável. Instituição de plano para a licença por invalidez, com comunicação regular entre a organização e o trabalhador. Retorno ao trabalho coordenado, planejado e facilitado por um coordenador designado que apoie a comunicação entre o líder, o trabalhador e a organização. Oferta de intervenções intensivas, multidisciplinares e baseadas em evidências. Apoio aos trabalhadores para obter os tratamentos disponíveis. Recomendação do treinamento dos líderes. Fornecimento de treinamento e conscientização em saúde mental para todos os trabalhadores para evitar o estigma. Avaliação do trabalho realizado pelo trabalhador e pelo supervisor.
		Intervenções clínicas sem modificações no trabalho e/ou orientação profissional.
Recomendações abordando os três segmentos da abordagem integrada no gerenciamento de problemas de saúde mental.	Memish *et al.*, 2017^(17)^	Recomendações para a organização minimizar os fatores de risco e promover fatores positivos no trabalho. Recomendações para prevenção do adoecimento mental direcionadas ao nível individual. Desenvolvimento de estilos de liderança, clima ou cultura organizacional positivos no local de trabalho. Detecção e tratamento dos problemas de saúde mental no local de trabalho.
		Falta de recursos e/ou pessoal capacitado para implementação das intervenções.
Recomendações para prevenir, detectar e gerenciar sinais precoce de adoecimento mental relacionado ao trabalho ou facilitar o retorno ao trabalho.	Nexo *et al.*, 2018^(18)^	Política de saúde mental em todos os níveis da organização (envolvimento dos trabalhadores, planejamento, recursos, funções e responsabilidades). Identificação e eliminação contínua e sistemática dos riscos psicossociais. Melhora da concepção do trabalho e adequação da pessoa-trabalho por meio de treinamento de habilidades e horários de trabalho rotativos em locais onde os riscos psicossociais não podem ser eliminados. Implementação de programas educacionais para conscientizar sobre a saúde mental e evitar o estigma associado ao adoecimento mental. Estímulo de fatores positivos do trabalho, como o apoio dos colegas e recompensas. Aumento da comunicação e competências relacionais dos líderes para evitar adoecimento mental. Avaliação da saúde mental dos trabalhadores. Estratégias para gerenciamento do estresse e dos problemas de saúde mental. Realização de aconselhamento no trabalho e adequações para trabalhadores com adoecimento mental. Estímulo de apoio/orientação entre os colegas de trabalho. Abordagem de retorno ao trabalho coordenada e multidisciplinar. Treinamento dos líderes para detectar sinais de adoecimento mental.
		Gestão inadequada do clima de trabalho, como assédio moral e conflitos no trabalho. Trabalho mal organizado com altas demandas, baixo controle e ameaças de violência por parte dos clientes.
